# Identification of Ochratoxin A Producing Fungi Associated with Fresh and Dry Liquorice

**DOI:** 10.1371/journal.pone.0078285

**Published:** 2013-10-21

**Authors:** Amanda Juan Chen, Dan Tang, Ying Qun Zhou, Bing Da Sun, Xiao Jin Li, Li Zhi Wang, Wei Wei Gao

**Affiliations:** 1 Institute of Medicinal Plant Development, Chinese Academy of Medical Sciences and Peking Union Medical College, Beijing, P.R. China; 2 China National Corporation of Traditional & Herbal Medicine, Beijing, P.R. China; 3 China General Microbiological Culture Collection Center, Institute of Microbiology, Chinese Academy of Sciences, Beijing, P.R. China; 4 Xinjiang Institute of Traditional Chinese Medicine and Ethnicdrug, Urumchi, P.R. China; Louisiana State University, United States of America

## Abstract

The presence of fungi on liquorice could contaminate the crop and result in elevated levels of mycotoxin. In this study, the mycobiota associated with fresh and dry liquorice was investigated in 3 producing regions of China. Potential toxigenic fungi were tested for ochratoxin A (OTA) and aflatoxin B1 (AFB1) production using liquid chromatography/mass spectrometry/mass spectrometry. Based on a polyphasic approach using morphological characters, β-tubulin and RNA polymerase II second largest subunit gene phylogeny, a total of 9 genera consisting of 22 fungal species were identified, including two new *Penicillium* species (*Penicillium glycyrrhizacola* sp. nov. and *Penicillium xingjiangense* sp. nov.). The similarity of fungal communities associated with fresh and dry liquorice was low. Nineteen species belonging to 8 genera were detected from fresh liquorice with populations affiliated with *P. glycyrrhizacola*, *P. chrysogenum* and *Aspergillus insuetus* comprising the majority (78.74%, 33.33% and 47.06% of total) of the community from Gansu, Ningxia and Xinjiang samples, respectively. In contrast, ten species belonging to 4 genera were detected from dry liquorice with populations affiliated with *P. chrysogenum*, *P. crustosum* and *Aspergillus terreus* comprising the majority (64.00%, 52.38% and 90.91% of total) of the community from Gansu, Ningxia and Xinjiang samples, respectively. Subsequent LC/MS/MS analysis indicated that 5 fungal species were able to synthesize OTA in vitro including *P. chrysogenum*, *P. glycyrrhizacola*, *P. polonicum*, *Aspergillus ochraceus* and *A. westerdijkiae*, the OTA concentration varied from 12.99 to 39.03 µg/kg. AFB1 was absent in all tested strains. These results demonstrate the presence of OTA producing fungi on fresh liquorice and suggest that these fungi could survive on dry liquorice after traditional sun drying. *Penicillium chrysogenum* derived from surrounding environments is likely to be a stable contributor to high OTA level in liquorice. The harvesting and processing procedure needs to be monitored in order to keep liquorice free of toxigenic fungi.

## Introduction

Liquorice, the root of the leguminous *Glycyrrhiza* plant species (*Glycyrrhiza uralensis* Fisch., *Glycyrrhiza inflate* Bat. and *Glycyrrhiza glabra* L.), is a popular botanical with a long history of cultivation and use in China. In Traditional Chinese Medicine, liquorice is one of the most frequently used herbs, which exerts antitussive, expectorant and antipyrotic functions and is often used to treat cough, pharyngitis, bronchitis and bronchial asthma [1]. In addition, liquorice is a common dietary supplement and its derivatives have been given Generally Recognized as Safe (GRAS) status in the USA in 1985 [2]. Likewise, liquorice and its derivatives are used as flavoring and sweetening agents in confectionery and other food products, such as beverages and chewing gum [3,4]. China is one of the largest liquorice producing regions. According to customs statistics in 2011, 3300 tons of liquorices were exported to Japan, Korea, Germany and the United States, among other countries. The wild plants of liquorice (*G. uralensis*, *G. inflate* and *G. glabra*) are primarily distributed in arid desert and grassland areas in Gansu, Ningxia, Xinjiang and Inner Mongolia in northwest China, among which *G. uralensis* is the most widely distributed variety [5]. 

Ochratoxin A (OTA) and Aflatoxin B1 (AFB1) are mycotoxins that cause adverse health effects in animals including teratogenicity, immunotoxicity, genotoxicity and mutagenicity [6,7]. The presence of ochratoxin A in liquorice was first reported in Germany by Bresch et al. and Majerus et al. [8,9]. Consequent studies confirmed widespread and high contamination of OTA in foods containing liquorice, sometimes with values exceeding 200 µg/kg [10,11]. In Spain, analysis of 30 samples of liquorice root, liquorice confectionery, liquorice block, and liquorice extract indicated that all samples contained OTA; the dry roots contained the highest OTA at levels of 1.4-252.8 µg/kg [12]. In China, 5 moldy liquorice samples were found to be contaminated with OTA at levels ranging 1.3-84.4 µg/kg [13]. Although liquorice and its derivatives were not main contributors to dietary intake of OTA, it cannot be excluded that liquorice confectionery may contribute to the level of exposure in consumers, in particular in children. In a worst case scenario, children with elevated intake of liquorice confectionary could reach the 8.94% tolerable weekly intake (TWI) [10]. In comparison with OTA, aflatoxin contamination of liquorice was found to be very low [14], the reason of this difference was still unknown.

OTA is mainly produced by several *Penicillium* and *Aspergillus* species, notably *Penicillium verrucosum* and *Aspergillus ochraceus*, but also *A. carbonarius* and *A. niger* species [15]. In a series of recent reviews and papers, more than 25 species of *Penicillium* were listed as OTA producers [16-18]. However Frisvad et al. [19] excluded most of them, and only accepted *P. verrucosum* and *P. nordicum* as major OTA producer in the Penicillia. With respect to liquorice, Chen et al. [20,21] found that *P. polonicum* and *P. chrysogenum* were the primary OTA contributors in moldy liquorice in China. However, the distribution pattern of these toxigenic fungi in regularly consumed liquorice is still unknown. In this study, both fresh and dry liquorice were collected from the major producing regions in China. The distribution of OTA producing fungi in Chinese liquorice and their pollution ways were studied in detail. 

## Materials and Methods

### Sample collection

Samples of fresh liquorice (*G. uralensis*) were collected from Gansu province (N 38°38’18’’, E 103°05’25’’, permitted by The Forestry Department of Gansu province), Ningxia province (N 37°46’55’’, E 107°24’18’’, permitted by The Forestry Department of Ningxia province) and Xinjiang province (N 42°17’59’’, E 86°24’18’’, permitted by The Forestry Department of Xinjiang province) in China. Twenty to 30 healthy wild liquorice roots were dug out carefully at each site and were gently shaken to remove superficial soil. Cleaned roots were then placed into sterile paper bags. Dry liquorice roots were collected from relevant local markets in Gansu, Ningxia and Xinjiang province. For each site, about 1 kg roots were collected from 5 different herbal stores and put into sterile paper bags. Samples from Gansu and Ningxia were whole dry roots which were dried in the sun at each respective locality while those from Xinjiang were herbal slices of roots, which were made from whole dry roots in the local processing factory. The temperatures of each sampling sites were 21.7°C, 20.0 °C, 17.1°C, and relative humidity 14%, 13%, 15% in Gansu, Ningxia and Xinjiang, respectively. All paper bags containing liquorice samples were transported to the lab in Beijing within 3 days of collection. 

### Isolation of fungi

Entire liquorice roots were cut into pieces with sterile scissors; herbal slices of roots were directly used for fungal isolation. Ten g of each sample was added to 90 ml sterile water and mixed. This mixture was then shaken on a rotary shaker for approximately 30 min and subjected to a series of ten-fold serial dilution to a final concentration of 10^-3^. Aliquots consisting of 1 ml of each dilution were spread (in quadruplicate) on Rose Bengal Chloramphenicol Agar (RBCA). One of the three sets of dilutions that averaged between 10 and 60 colonies per plate was selected for enumeration. The results of quadruplicate plating were expressed as the average CFU/g. All of the plates were incubated at 25 °C for 7 to 10 days and were subsequently stored at 4 °C for future colony isolation and identification.

### Identification of fungi

Followed by preliminary morphological identification, every fungal colony was transferred and re-streaked onto Malt Extract Agar (MEA). With respect to new *Penicillium* species, colonies on Czapek Yeast Autolysate Agar (CYA) and Yeast Extract Sucrose Agar (YES) cultivated at 25 °C for 7 days were also compared and described. Colony colour was assessed according to The Methuen Handbook of Colour by Kornerup and Wanscher [22]. Other macroscopical and microscopic morphological observations (eg. colony texture, conidiophore and conidia characteristics) were made according to proper guides [23-26]. To verify the results of morphological characterization and identification of fungi, the β-tubulin gene and the second largest RNA polymerase II subunit (RPBII) gene were PCR amplified and sequenced. In total, 126 isolates affiliated with *Aspergillus* and *Penicillium* were subjected to PCR amplification of the β-tubulin gene using the primer pair Bt2a 5'-GGTAACCAAATCGGTGCTGCTTTC-3' and Bt2b 5'-ACCCTCA GTGTAG TGACCCTTGGC-3' [27]. A part of RPBII gene were amplified from these strains with the primer pair RPB2-5F_Pc 5’- GATGACCGTGACCACTTCGG-3’ and RPB2-7CR_Pc 7CR 5’-CCCATGGCTTGTTTGCCCAT-3’ [28]. With respect to fungal species other than *Penicillium* and *Aspergillus* spp., ITS gene was amplified by using the primers ITS4 5'- TCCTCCGCTTATTGATATGC -3' and ITS5 5'- GGAAGTAAAAGTCGTAACAAGG -3' [29]. Basic Local Alignment Search Tool (BLAST) was used to identify the closest affiliated sequence in the GenBank/NCBI dataset.

### Phylogenetic tree construction

Phylogenetic analyses were conducted in order to better understand the evolutionary history of isolates using the obtained partial β-tubulin and RPBII genes. Sequences of selected strains along with reference sequences obtained from GenBank (Table 1) were aligned using Clustal X [30]. Alignment was manually trimmed at the N and C terminals to delete non-overlapping alignment regions with the MEGA5 program [31]. Phylogenetic analyses using the neighbor-joining method [32] was performed with the same program. The neighbor-joining tree was constructed specifying 1) the Kimura 2-parameter model, 2) transitions and transversions and 3) with pairwise deletion of gaps. The phylogeny was subjected to 1000 bootstrap replicates. All phylograms were rooted with gene sequences from *A. westerdijkiae*. 

**Table 1:** Strains used in phylogenetic analysis.

Taxon name	Collection No.	Origin	GenBank Accession number	
			ß-tubulin	RPBII
*Penicillium aethiopicum*	CBS484.84 T	Grain, Ethiopia	AY495983	JF909940
*P. aurantiogriseum*	CBS 324.89 T	Unknown	AY674296	JN406573
	CBS 792.95	Apple juice production plant, Denmark	AY674298	JN985381
	CBS 642.95	Chicken feed, Denmark	AY674297	JN985380
*P. chrysogenum*	CBS 306.48 T	Cheese, USA	AY495981	JF909937
	NRRL 807 T	Cheese, USA	JF909943	JF909925
	CBS 355.48 T	Decaying branch, Norway	JF909948	JF909930
	CGMCC3.15262(G1433)	Liquorice, China, Gansu	KF021541	KF021564
	CGMCC3.15263(G3311)	Liquorice, China, Gansu	KF021539	KF021552
	CGMCC3.15266(G6523)	Liquorice, China, Gansu	KF021542	KF021551
	CGMCC3.15265(ND3341)	Liquorice, China, Ningxia	KF021545	KF021565
*P. confertum*	CBS171.87 T	Cheek pouch, USA	AY674373	JF909934
*P. cyclopium*	CBS 144.45 T	Fruit, Norway	AY674310	JN985388
	CBS 477.84	Grain, Denmark	AY674309	JN985393
	CBS 101136	Harness, Saudi Arabia	AY674308	JN985383
*P. dipodomyis*	CBS110412 T	Cheek pouch, USA	AY495991	JF909932
	CBS 170.87	Cheek pouch, USA	AY495989	JX996707
*P. flavigenum*	CBS 419.89 T	Flour, Denmark	AY495993	JF909939
	CBS110411	Barley, Canada	JX996830	JX996694
*P. freii*	CBS 794.95	Chicken feed (cereal), Denmark	AY674290	JN985430
	CBS 112292	Barley, Denmark	AY674292	JN985397
*P. gladioli*	CBS 332.48 T	Corm of Gladiolus sp., USA	AY674287	JN406567
	CBS815.70	Corm of Gladiolus sp., India	AY674289	
*P. glycyrrhizacola* sp. nov.	CGMCC3.15271(G4432) T	Liquorice, China, Gansu	KF021538	KF021554
	CGMCC3.15269(G1411)	Liquorice, China, Gansu	KF021540	KF021556
	CGMCC3.15273(74212) T	Liquorice, China, Xingjiang	KF021543	KF021553
	CGMCC3.15270(G2312)	Liquorice, China, Gansu	KF021546	KF021555
*P. melanoconidium*	CBS 640.95	*Panicum miliaceum* imported to Denmark	AY674303	JN985431
	CBS 218.90	*Hordeum vulgare*, Denmark	AY674302	JN985404
*P. mononematosum*	CBS172.87 T	Heavily moulded seed, USA	AY495997	JF909935
*P. nalgiovense*	CBS 352.48 T	Ellischauer cheese, Czechoslovakia	AY495999	JF909938
	CBS 109610	Unknown	JX996825	JX996689
*P. neoechinulatum*	CBS 101135 T	Cheek pouch, USA	AY674299	JN985406
	CBS 110343	Seed cache, USA	AY674300	JN985409
*P. nordicum*	CBS 109538	Fish feed, Denmark	AY674314	
	CBS 109537	Jam, Japan	AY674315	
	CBS 109541	Lumpsucker, Denmark	AY674316	
	CBS 112573	Salami, Italy	AY674317	
	CBS 110770	Sausage, Germany	AY674318	
*P. persicinum*	CBS 111235 T	Soil, China	AY495982	JF909933
*P. polonicum*	CBS 222.28 T	Soil, Poland	AY674305	JN406609
	CBS 101479	Foods, Bulgaria	AY674306	JN985410
	CGMCC3.15264(G3323)	Liquorice, China, Gansu	KF021548	KF021558
	CGMCC3.15272(G5314)	Liquorice, China, Gansu	KF021549	KF021559
	CGMCC3.15221(16524)	Liquorice, China, Jiangxi	HM347088	KF021562
	CGMCC3.15222(23226)	Liquorice, China, Henan	HM347089	KF021563
*P. tricolor*	CBS635.93 T	*Triticum aestivum*, Canada	AY674313	JN985422
	CBS 637.93	*Triticum aestivum*, Canada	AY674312	JN985423
*P. verrucosum*	CBS 603.74	Unknown, Belgium	AY674323	JN121539
	CBS 115508	Unknown	AY674324	
*P. viridicatum*	CBS 390.48 T	Air, USA	AY674295	JN985429
	CBS 109826	Cereal, Bulgaria	AY674294	JN985426
*P. xingjiangense* sp. nov.	CGMCC3.15274(84513)	Liquorice, China, Xingjiang	KF021544	KF021557
*Aspergillus ochraceus*	CGMCC3.15267(74124)	Liquorice, China, Xingjiang		KF021560
*A. westerdijkiae*	CGMCC3.15268(84424)	Liquorice, China, Xingjiang	KF021547	KF021561

### Nomenclature

The electronic version of this article in Portable Document Format (PDF) in a work with an ISSN or ISBN will represent a published work according to the International Code of Nomenclature for algae, fungi, and plants, and hence the new names contained in the electronic publication of a PLOS ONE article are effectively published under that Code from the electronic edition alone, so there is no longer any need to provide printed copies. In addition, new names contained in this work have been submitted to MycoBank from where they will be made available to the Global Names Index. The unique MycoBank number MB804682 and MB804683 can be resolved and the associated information viewed through any standard web browser by appending the MycoBank number contained in this publication to the prefix http://www.mycobank.org/MB/. The online version of this work is archived and available from the following digital repositories: PubMed Central, LOCKSS etc.

### OTA and AFB1 determination of fungal strains by liquid chromatography/mass spectrometry/mass spectrometry (LC/MS/MS)

Fungal strains were grown on sterile rice media (20 g rice; 20 ml water, sterilize for 30 min) for 30 days at 25 °C. After incubation, all cultures were treated at 60 °C for three days to dry the mycelial mat and then finely grounded using a grinder. About 20 g mat material and 100 ml methanol-water (80/20) was mixted and extracted using ultrasonography for 45 min and filtered. The filtrate was purified by solid phase extraction (SPE) (NERCB-SPE, 100 mg/3 ml, Beijing, China). LC/MS/MS analysis was performed with an Agilent 1200 series high-performance liquid chromatograph (HPLC) (Palo Alto, CA, USA) interfaced to a 3200Q Trap triple-quadrupole linear ion-trap MS/MS system (Applied Biosystems, Foster City, CA, USA). The sample was separated using a XbridgeTM C18 column (150 × 2.1 mm, 3.5 µm) (Waters, Milford, Massachusetts, USA) with acetonitrile- water containing 5 mM ammonium acetate. OTA and AFB1 were detected with ESI in positive mode. The collision energy (CE) was 40.0 V. The MRM model was used for quantitative calculation. The ion pairs at m/z 404/358 and m/z 313/285 were employed to analyze OTA and AFB1, respectively. The limits of detection (LOD) and the limits of quantification (LOQ) were less than 0.024 and 0.095 µg/kg, respectively [33].

### Statistical analysis

Isolation frequency was determined as the number of isolates of one species divided by the total number of isolates obtained. The species diversity was estimated using the Shannon-Wiener index (*H*´) according to the formula: $H'\text{=-}\sum_{i=1}^{n}P_{i}1nP_{i}$[34], where *P*_*i*_ is the proportion of the *i*th species and *n* is the number of species at the site. The Bray-Curtis similarity coefficient (*C*) was used to estimate the similarity of fungal communities as *C* = 2*w*/(*a*+*b*) [35], where *w* is the sum of the lesser counts of each species common to both sites, *a* and *b* were the sum of all isolates obtained in each site.

The software SPSS 15.0 was used for the statistical data analysis. Analyses were made of fungal colony counts of different regions by standard χ^2^ tests. Multiple pairwise comparisons were performed using the Table procedure of SPSS software and the Bonferroni method was used to adjust *P*-values.

## Results

### Phylogeny and Taxonomy of Fungal Species

**Figure 1:**
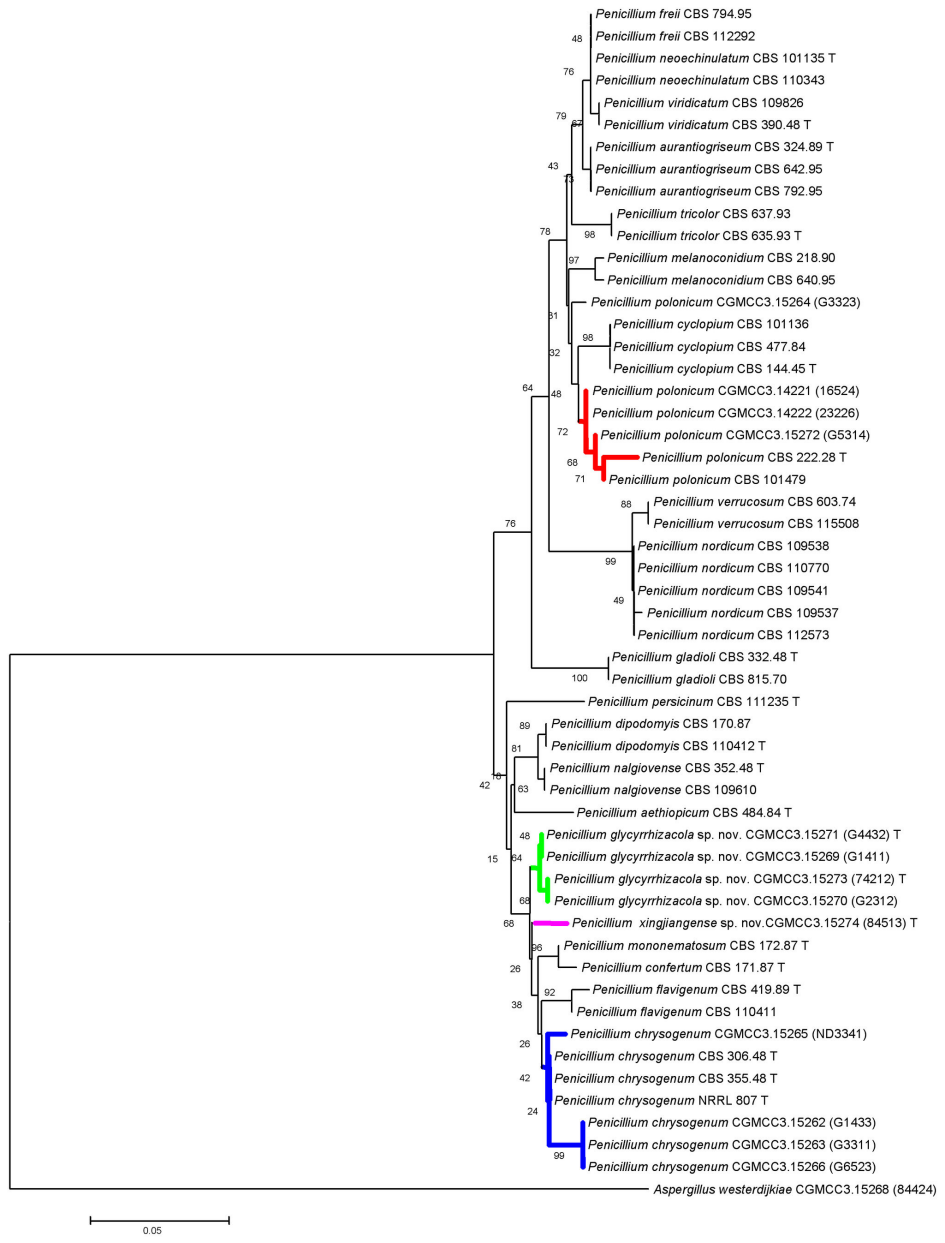
Neighbor-joining tree inferred from partial β-tubulin genes showing the relationships among members of *Penicillium* subgenus *Penicillium* sect. Viridicata and sect. Chrysogena. The tree is rooted with the β -tubulin gene from *Aspergillus westerdijkiae*.

**Figure 2:**
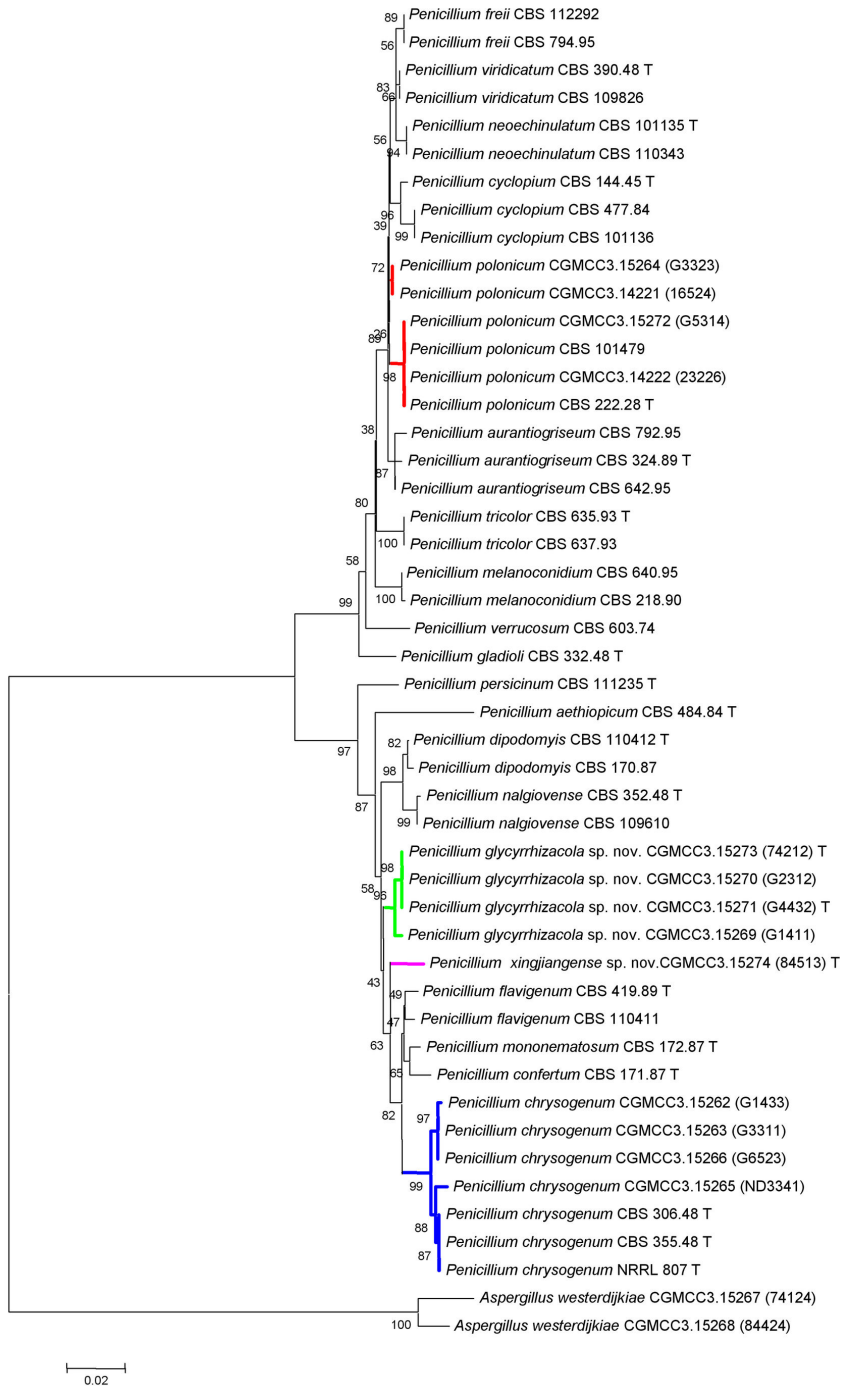
Neighbor-joining tree inferred from partial RPBII genes showing the relationships among members of *Penicillium* subgenus *Penicillium* sect. Viridicata and sect. *Chrysogena*. The tree is rooted with the RPBII gene from *Aspergillus westerdijkiae*.

All fungal strains were identified according to morphological and molecular characteristics. With respect to confusing species and potential toxigenic species, twelve β-tubulin and 15 RPBII sequences were included in the analysis. In addition, forty-one and 32 sequences recovered from GenBank that were associated with *Penicillium* subgenus *Penicillium* sect. Viridicata and sect. Chrysogena, respectively, were included as references. Figure 1 indicates that members of the sect. Viridicata and Chrysogena formed two separate clades. *Penicillium polonicum* strain CGMCC3.15272 (G5314) clustered with type species CBS 222.28. *Penicillium polonicum* strain CGMCC3.15264 (G3323) was excluded from this clade, but subsequent morphological observations (blue green conidia on CYA and YES) suggests that it should be included in this lineage. In clade Chrysogena, described *P. chrysogenum* strains formed an independent lineage that clustered with type species CBS 306.48. Two new branches were separated from other known species with rather high bootstrap value. Combined with detailed morphological observation, they were identified as two new species, namely *Penicillium glycyrrhizacola* sp. nov. and *Penicillium xingjiangense* sp. nov.. The phylogenetic position of isolates when the RPBII sequence was used is in full agreement with that when β-tubulin sequence was used. (Figure 2)

**Figure 3:**
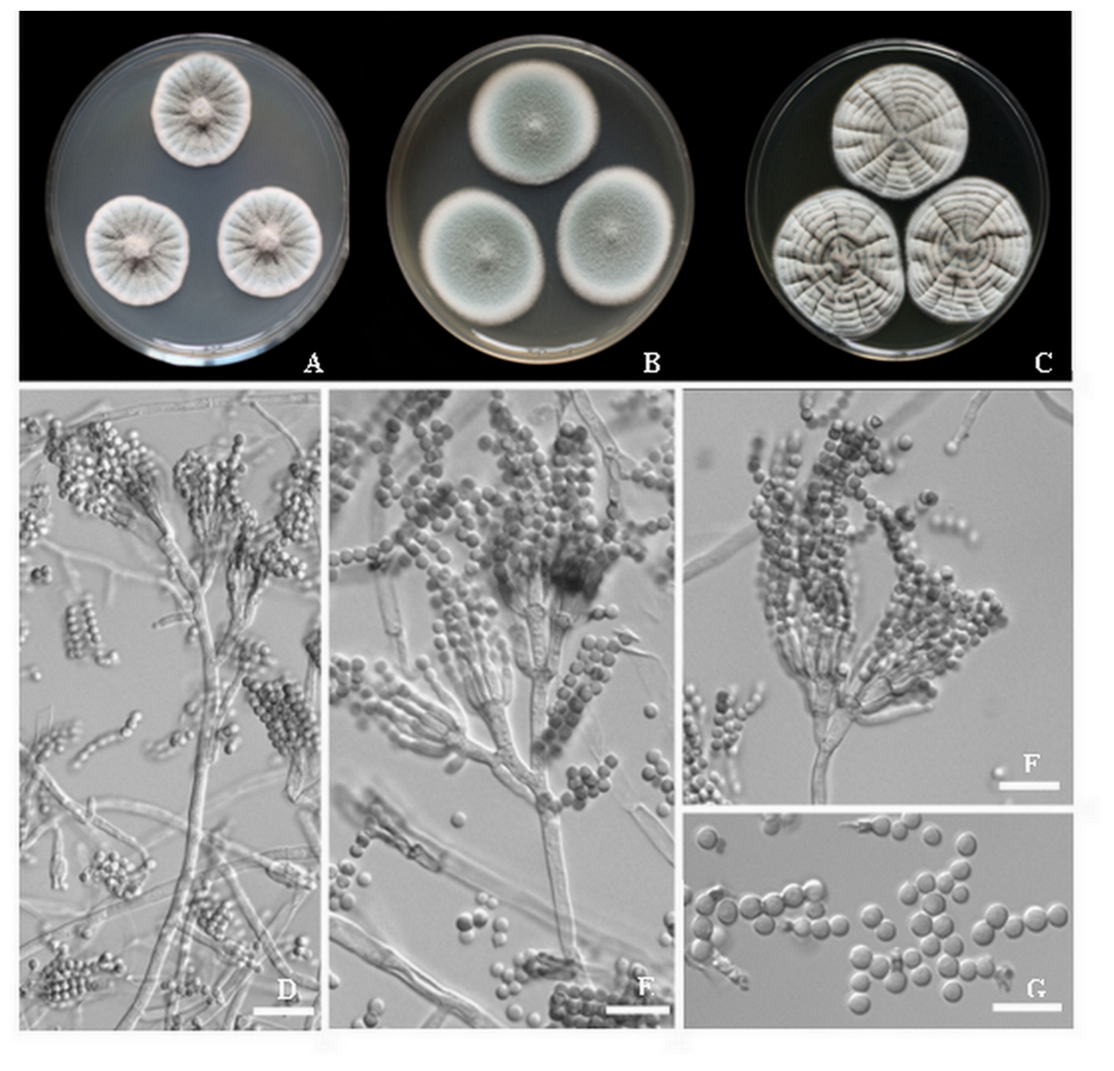
*Penicillium glycyrrhizacola* sp. nov. A-C. 7-day old colonies on A. CYA, B. MEA, C. YES, D-F. Conidiophores. **G**. Conidia. Scale bar = 10 µm.

*Penicillium glycyrrhizacola* Chen, Sun & Gao, sp. nov. Figure 3.

Mycobank no.: MB804682

Colonies on CYA at 25°C reaching 23-34 mm diam. in 7 d, floccose, radially furrowed; mycelium white to grayish green (25B3), exudates rarely present, clear, reverse grayish yellow (33C) to white at center.

Colonies on MEA at 25°C reaching 23-34 mm diam. in 7 d, floccose to velutinous; mycelium grayish green (25B3); sporulation heavy on entire surface of colonies, reverse white.

Colonies on YES at 25°C reaching 31-38 mm diam. in 7 d, velvety, crater-like or convoluted at center, radially furrowed, mycelium white to grayish green (25C3), reverse grayish yellow (33C) to white.

Conidiophores borne from surface or subsurface hyphae, stipes commonly 150-300 ×2.5-3.5 µm, ter- and quarter verticillate, divergent rami born from aerial and subsurface hyphae; rami 13-20×3.0-4.0 µm; metulae in verticals of 3-5, short and appressed, 9-12×2.5-3.5 µm, phialides 4-7 per vertical, ampilliform 8-10×2.5-3 µm. Conidia ellipsoidal to subspheroidal, smooth walled, 2.2-2.6 × 2.5-3.2 µm.

Habitat: Liquorice, China

Holotype: China, Gansu, from liquorice, CGMCC3.15271 (G4432), specimen deposited in Herbarium Mycologicum Academiae Sinicae HMAS 244707; *Paratype*: China, Xingjiang, from liquorice, CGMCC3.15273 (74212), HMAS 244708.

Distinguishing characteristics: *Penicillium glycyrrhizacola* shares many characters in common with *P. chrysogenum*, but differs by the grayish green colony on CYA and MEA, rarely present clear exudates and absent soluble pigment.

**Figure 4:**
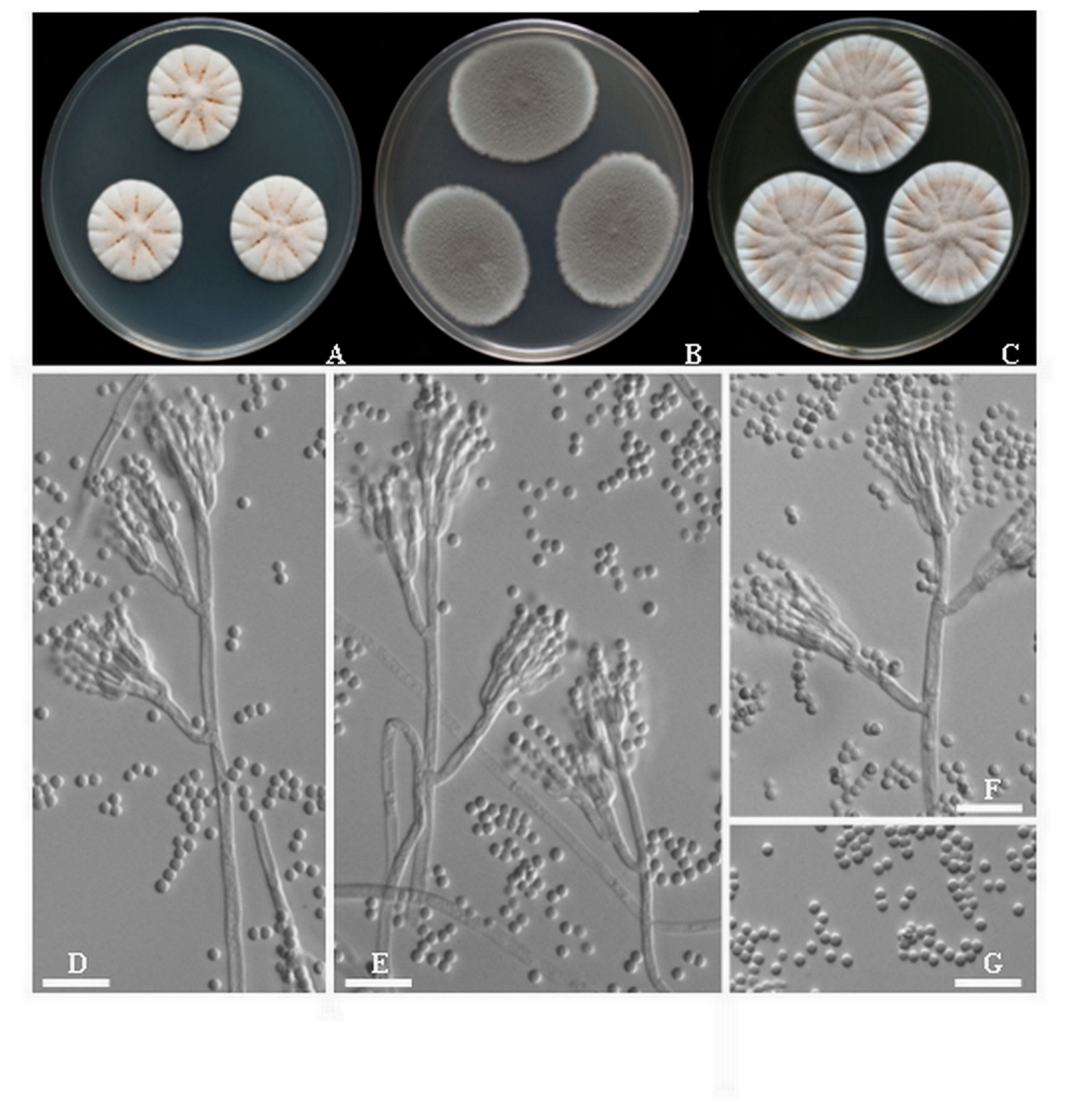
*Penicillium xingjiangense* sp. nov. A-C. 7-day old colonies on A. CYA, B. MEA, C. YES, D-F. Conidiophores. **G**. Conidia. Scale bar = 10 µm.

*Penicillium xingjiangense* Chen, Sun & Gao, sp. nov. Figure 4.

Mycobank no.: MB804683

Colonies on CYA at 25°C reaching 29-32 mm diam. in 7 d, floccose, radially furrowed; mycelium white to pale yellow (4A3), exudates clear, reverse grayish yellow (3C3).

Colonies on MEA at 25°C reaching 20-24 mm diam. in 7 d, floccose to velutinous; mycelium grayish green (4B3) at centre, turquoise white (24A2) towards white at margin; sporulation heavy on entire surface of colonies, reverse white. 

Colonies on YES at 25°C reaching 39-45 mm diam. in 7 d, velvety, radially furrowed, mycelium orange grey (6B2), reverse pale yellow (1A3).

Conidiophores borne from surface or subsurface hyphae, stipes commonly 100-300 ×3.0-3.2 µm, ter- and quarter verticillate, divergent rami born from aerial and subsurface hyphae; rami 21-30×3.0-3.2 µm; metulae in verticals of 2-5, thin and appressed, 9-13×2.0-3.0 µm, phialides 5-7 per vertical, ampilliform 8-10×2-3 µm. Conidia mostly globose, smooth walled, 2.0-2.2 × 2.8-3.2 µm.

Habitat: Liquorice, China

Holotype: China, Xingjiang, from liquorice, CGMCC3.15274 (84513), specimen deposited in Herbarium Mycologicum Academiae Sinicae, HMAS 244709.

Distinguishing characteristics: *Penicillium xingjiangense* is closely relate to *P. confertum* and *P. mononematosum*, but differs by the white to pale yellow colony on CYA and thin metulae.

### Comparison of fungal community on fresh and dry liquorice

Total fungal counts ranged from 300 to 3400 CFU/g on fresh liquorice sampled from 3 production areas; nineteen species of fungi belonging to 8 genera were detected among these CFUs (Table 2). The fungal community associated with fresh liquorice showed high species diversity with the Shannon-Weiner index (*H*’) of 1.971. *Penicillium* and *Aspergillus* were the two most predominant genera detected in association with fresh liquorice, representing 49.57% and 24.98% of the total CFUs respectively. *Penicillium glycyrrhizacola*, *P. chrysogenum* and *A. insuetus* were the most common species on fresh liquorice from Gansu, Ningxia and Xinjiang province; *Penicillium chrysogenum* was the only species which was detected from all three of the production areas. In contrast, a total of 2200 to 9375 CFU were obtained per g dry liquorice. Among these CFUs, ten species belonging to 4 genera were detected. The fungal community associated with dry liquorice showed low species diversity as evinced by a Shannon-Weiner index (*H*’) as 1.755 when compared to 1.971 associated with the fresh liquorice. Like fresh liquorice, *Penicillium* and *Aspergillus* were the two most abundant genera detected, representing 73.54% and 14.26% of the CFUs. *P. chrysogenum* was the most common species on dry liquorice from Gansu and could be detected in both Ningxia and Xinjiang samples while with relative low frequency. Seven fungal species could be detected in association with both fresh and dry liquorice which including *A. westerdijkiae*, *Eurotium amstelodami*, *Penicillium aurantiogriseum*, *P. chrysogenum*, *P. crustosum*, *P. glycyrrhizacola* and *P. polonicum*. The similarity of fungal communities between fresh and dry liquorice was 22% (Bray-Curtis index). Among the three production areas examined, the most similar dry and fresh liquorice fungal communities were found in Gansu, with a Bray-Curtis index of 31.78%, followed by Ningxia (Bray-Curtis index = 6.67%) and Xinjiang (Bray-Curtis index = 4.46%).

**Table 2:** Frequency (%) of fungi associated with fresh and dry liquorice of 3 production areas in China.

Fungal species	fresh liquorice				dry liquorice		
	Gansu	Ningxia	Xinjiang		Gansu	Ningxia	Xinjiang
*Acremonium kiliense*			0.74				
*Actinomucor elegans*			0.74				
*Aspergillus dimorphicus*			9.56				
*Aspergillus insuetus*			47.06				
*Aspergillus ochraceus*					1.33		
*Aspergillus terreus*							90.91
*Aspergillus westerdijkiae*			7.35		1.33		
*Cladosporium cladosporioides*			1.47				
*Eurotium amstelodami*		33.33			1.33		
*Eurotium repens*	0.92		22.79				
*Metarhizium anisopliae*	0.18						
*Mucor racemosus*						42.86	
*Penicillium aurantiogriseum*	0.18				4.00		
*Penicillium chrysogenum*	7.58	33.33	3.68		64.00	4.76	9.09
*Penicillium citrinum*			3.68				
*Penicillium coprophilum*			0.74				
*Penicillium crustosum*	5.36	16.67				52.38	
*Penicillium glycyrrhizacola*	78.74		1.47		16.00		
*Penicillium griseofulvum*	1.11						
*Penicillium polonicum*	5.91				12.00		
*Penicillium xingjiangense*			0.74				
*Talaromyces flavus*		16.67					
Total counts (CFU/g)	2705	300	3400		9375	4200	2200

### OTA and AFB1 production by selected strains

**Figure 5:**
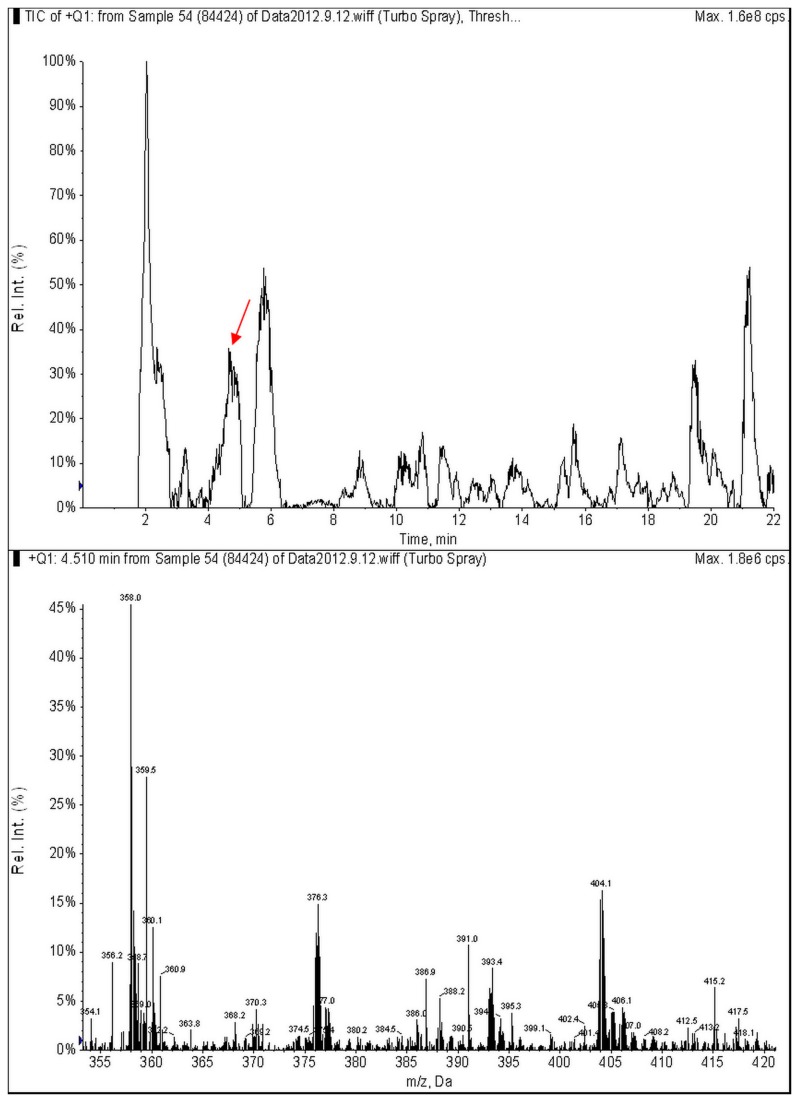
LC-MS/MS chromatography for OTA positive strain CGMCC3.15268 (84424) with Q1 mode. Ions at m/z 404 and m/z 358 were selected as characteristic signals of OTA, while the ion at m/z 406 containing ^37^Cl was a reference.

Among the 21 fungal species obtained in this study, 5 species (*P. polonicum, P. chrysogenum, P. crustosum, A. ochraceus* and *A. westerdijkiae*) were classified as potentially being toxigenic [16–18,21]. Accordingly, 21 strains belonging to these five species and 2 new species *P. glycyrrhizacola* and *P. xingjiangense* were screened for OTA and AFB1 production. OTA could be detected in 6 strains belong to 5 species (Table 3). A chromatogram with the mass spectrum from an OTA positive sample is depicted in Figure 5. As shown in Table 3, *P. chrysogenum*, *P. glycyrrhizacola*, *P. polonicum*, *A. ochraceus* and *A. westerdijkiae* could produce OTA. The OTA concentration varied among the isolates from 12.99 to 39.03 µg/kg. AFB1 was absent in all tested strains.

**Table 3:** OTA and AFB1 production by fungal species*.

Sample No.	Taxon name	Strain No.	Substratum	Origin	OTA(µg/kg)	AFB1(µg/kg)
1	*Penicillium chrysogenum*	CGMCC3.15262(G1433)	fresh liquorice	Gansu province, China	n.d.	n.d.
2		CGMCC3.15263(G3311)	fresh liquorice	Gansu province, China	n.d.	n.d.
3		CGMCC3.15265(ND3341)	fresh liquorice	Ningxia province, China	26.62	n.d.
4		CGMCC3.15266(G6523)	dry liquorice	Gansu province, China	n.d.	n.d.
5	*P. glycyrrhizacola*	CGMCC3.15271(G4432) T	fresh liquorice	Gansu province, China	n.d.	n.d.
6		CGMCC3.15269(G1411)	fresh liquorice	Gansu province, China	n.d.	n.d.
7		CGMCC3.15270(G2312)	fresh liquorice	Gansu province, China	n.d.	n.d.
8		CGMCC3.15273(74212) T	fresh liquorice	Xinjiang province, China	30.44	n.d.
9	*P. polonicum*	CGMCC3.15264(G3323)	fresh liquorice	Gansu province, China	12.99	n.d.
10		CGMCC3.15272(G5314)	dry liquorice	Gansu province, China	39.03	n.d.
11	*Aspergillus ochraceus*	CGMCC3.15267(74124)	fresh liquorice	Xinjiang province, China	26.48	n.d.
12	*A. westerdijkiae*	CGMCC3.15268(84424)	fresh liquorice	Xinjiang province, China	17.26	n.d.

* Only OTA positive species were listed here

## Discussion

This study provides the first comprehensive evidence for the presence of OTA producing fungi on fresh and dry liquorice from China and provides a comprehensive assessment of the associated communities. Two new *Penicillium* species (*Penicillium glycyrrhizacola* sp. nov. and *Penicillium xingjiangense* sp. nov.) were identified and characterized genetically and morphologically. Importantly, one of these species, *P. glycyrrhizacola*, was the dominant fungus detected on fresh liquorice sampled from Gansu province. 

Fungal contamination on dry liquorice has been the subject of many investigations [36,37], however OTA producing fungi on liquorice has rarely been reported. Chen et al. [21] investigated toxigenic fungi associated with OTA contaminated and moldy liquorice. According to their finding, the predominant fungal species differed based on the location of liquorice sampling; only *P. polonicum* and *P. chrysogenum* contributed to high OTA levels in the moldy liquorice samples that were tested. Moldy liquorice resulting from improper storage will not be processed or sold to customers under normal circumstances. Thus, identifying OTA producing fungi associated with healthy dry liquorice was the focus of this study. Here, we report that *P. chrysogenum* was the most common species associated with dry liquorice sampled from 3 production regions in China and subsequent LC/MS/MS analysis proved the ability of this strain to produce OTA. Since *P. chrysogenum* was detected in association with both healthy dry and OTA positive moldy liquorice, we suggest that *P. chrysogenum* is a stable contaminant on liquorice and is one of main contributors of OTA contamination. 

In addition to *P. chrysogenum*, other OTA producing fungi associated with liquorice contained *P. glycyrrhizacola*, *P. polonicum*, *A. ochraceus* and *A. westerdijkiae* which account for 18.22% of total fungal species on dry liquorice. The OTA producing ability of *P. glycyrrhizacola* was reported for the first time in this study. *Penicillium polonicum* has previously been reported as an OTA producer that is associated with moldy liquorice [21]. *Aspergillus ochraceus* and *A. westerdijkiae* were responsible for OTA in feeds, bee pollen, cocoa and smoked red pepper [38-41]. Although the quantity of these OTA producing fungi was lower than that of *P. chrysogenum* on dry liquorice, they could become predominant under proper environmental conditions, and thus may be responsible for the high OTA level in liquorice. It’s worth noting that the OTA producing abilities of *P. chrysogenum* and *P. polonicum* were questioned by some researchers, only *P. verrucosum* and *P. nordicum* were accepted as OTA producers among the Penicillia [19]. *Penicillium verrucosum* and *P. nordicum* are mainly distributed in cereal and meat [42-44] and have never been detected in association with liquorice [20,21,36,37]. This study verified that a proportion of the tested *P. chrysogenum* strains (one of four tested strains) could produce OTA. In comparison, none of the isolated fungi were found to produce AFB1 in this study. 

Low fungal community similarity was found between fresh and dry liquorice, *Penicillium* spp. and *Aspergillus* spp. were the dominant fungi encountered. Similarly, Efuntoye [45] reported remarkable differences between the mycobiota obtained on fresh and dry plant parts; however, *Rhizopus stolonifer*, *Absidia corymbifera*, *Helminthosporium* sp., *Pichia fermentans* and *Trichosporon* sp. were found to be associated primarily with the fresh plant while *Aspergillus*, *Penicillium*, *Fusarium*, *Rhizopus* and *Mucor* were found to be more commonly associated with dry plant samples. The possible reason for this observation is that the fresh plant parts used in his research included seeds, leaves, fruits and stem barks; the normal mycobiota associated with fresh plant was unlikely to be able to exist after it was sun dried. With respect to liquorice, fresh roots were dug up from soil and it is hard to remove the *Penicillium* and *Aspergillus* fungal spores along with soil by traditional washing methods. These kinds of fungi may survive for a long period of time after drying [46,47]. So liquorice may be more easily contaminated by fungi and mycotoxins due to the part of the plant that is utilized and the rough way in which it is processed. 

Fewer fungal species were found in association with dry liquorice from Xinjiang, species of *Penicillium* were rarely detected and *A. terreus* was predominant. Meanwhile the lowest fungal community similarity was found between fresh and dry liquorice sampled from Xinjiang. This partly because the dry liquorices from Gansu and Ningxia were whole roots, which were dried directly after harvest while samples from Xinjiang were liquorice slices, which were made from whole dry roots in the local processing factory. The normal processing procedure included washing, cutting and drying. Importantly, it is possible that the primary mycobiota present on raw roots may have been removed and replaced by fungi present in the processing factory. Compared with whole liquorice roots, slices of liquorice are more often used in hospital and pharmacy settings. According to our data, liquorice slices appear to be less contaminated by fungi and thus may support the continued use of this liquorice processing technique over liquorice roots in these settings. 

From this study it can be concluded that fungal contamination constitutes a potential health hazard in consumption of liquorice. Ariño et al. [48] indicated that the OTA in licorice and derived products was unaffected by sorting or washing, whereas peeling the roots significantly reduced OTA contents by more than 50%. However, this method of processing is typically viewed as being cost-prohibitive. According to our findings, *P. chrysogenum*, *P. glycyrrhizacola*, *P. polonicum* and *A. westerdijkiae* present on fresh liquorice could survive after primary sun drying and were main OTA contributors on dry liquorice. The information determined in this study will further efforts to identify more targeted fungicidal methods to help keep liquorice crops free from these toxigenic fungi. 
